# Single-Cell RNA Sequencing Profiles Identify Important Pathophysiologic Factors in the Progression of Diabetic Nephropathy

**DOI:** 10.3389/fcell.2022.798316

**Published:** 2022-05-10

**Authors:** Xi Lu, Li Li, Luolan Suo, Ping Huang, Hongjie Wang, Su Han, Mingming Cao

**Affiliations:** ^1^ Department of Gastroenterology, The First Affiliated Hospital of Harbin Medical University, Harbin, China; ^2^ Department of Endocrinology, The First Affiliated Hospital of Harbin Medical University, Harbin, China; ^3^ Department of Endocrinology, The Fourth Affiliated Hospital of Harbin Medical University, Harbin, China; ^4^ Department of Parasitology, Harbin Medical University, Harbin, China

**Keywords:** diabetic nephropathy, single-cell RNA sequencing, marker genes, immune cells, mTOR pathway

## Abstract

**Objective:** Single-cell RNA sequencing (scRNA-seq) analyses have provided a novel insight into cell-specific gene expression changes in diseases. Here, this study was conducted to identify cell types and pathophysiologic factors in diabetic nephropathy.

**Methods:** Single-cell RNA sequencing data of three human diabetic kidney specimens and three controls were retrieved from the GSE131882 dataset. Following preprocessing and normalization, cell clustering was presented and cell types were identified. Marker genes of each cell type were identified by comparing with other cell types. A ligand–receptor network analysis of immune cells was then conducted. Differentially expressed marker genes of immune cells were screened between diabetic nephropathy tissues and controls and their biological functions were analyzed. Diabetic nephropathy rat models were established and key marker genes were validated by RT-qPCR and Western blot.

**Results:** Here, 10 cell types were clustered, including tubular cells, endothelium, parietal epithelial cells, podocytes, collecting duct, mesangial cells, immune cells, distal convoluted tubule, the thick ascending limb, and proximal tubule in the diabetic kidney specimens and controls. Among them, immune cells had the highest proportion in diabetic nephropathy. Immune cells had close interactions with other cells by receptor–ligand interactions. Differentially expressed marker genes of immune cells EIF4B, RICTOR, and PRKCB were significantly enriched in the mTOR pathway, which were confirmed to be up-regulated in diabetic nephropathy.

**Conclusion:** Our findings identified immune cells and their marker genes (EIF4B, RICTOR, and PRKCB) as key pathophysiologic factors that might contribute to diabetic nephropathy progression.

## Introduction

Diabetic nephropathy represents a microvascular complication of type 1 and 2 diabetes, which may rapidly progress to an end-stage renal disease without treatment due to having no clinical symptoms at an early stage (Sun et al., 2021). It occupies 40% of patients who require renal replacement therapy (Lu et al., 2020). The morphological and ultrastructural alterations contain glomerular basement membrane thickening, mesangial matrix expansion, glomerular hyperfiltration as well as tubular interstitial fibrosis (Wheeler et al., 2021). Despite microalbuminuria as a biomarker for early diabetic nephropathy, it possesses low sensitivity and specificity in predicting the risks of diabetic nephropathy (Lee et al., 2021). Moreover, renal biopsy is still the gold standard for diagnosing diabetic nephropathy (Chen et al., 2021). However, it is an invasive examination with a few adverse events like infection and hemorrhage (Li et al., 2021). Furthermore, it can hardly proceed continuously as the renal disease progresses and has a high probability of sampling errors. Hence, it is of importance to investigate non-invasive and sensitive biomarkers for predicting diabetic nephropathy progression. Diabetic nephropathy is a multi-factorial disease, which has the features of the complex interactions of hemodynamic and metabolic factors, such as hyperglycemia, advanced glycation end-product, and activation of the renin-angiotensin-aldosterone system (Du et al., 2021). Thus, the current management of diabetic nephropathy places emphasis on a strict control of blood sugar, blood pressure, and blood lipids (Ahola et al., 2021). Nevertheless, these strategies cannot protect against chronic kidney diseases. Hence, it is extremely important to find new treatments for diabetic nephropathy.

Traditional RNA sequencing (RNA-seq) may allow detecting gene expression alterations between cell populations via differential expression analyses. Nevertheless, RNA-seq cannot find genes that cause differences between cells since RNA-seq specimens are retrieved from a mixture of cells (Yip et al., 2019). Single-cell RNA sequencing (scRNA-seq) has been widely applied for studying gene expressions at the level of an individual cell. The sensitivity, accuracy, and efficiency of scRNA-seq have been making much progress in recent years (Ding et al., 2020). Through single-cell profiles in a mix of cell populations, scRNA-seq shows favorable advantages over RNA-seq such as dissection of heterogeneity in cell populations and exploration of rare cell types related to diseases (Hafemeister and Satija, 2019). By scRNA-seq of glomerular cells, dynamic changes in gene expression have been found in experimental diabetic kidney diseases (Fu et al., 2019). Here, this study identified specific cell types and marker genes in diabetic nephropathy. Immune cells were considered as a determinant of diabetic nephropathy. In diabetic nephropathy rat models, we verified the expression of marker genes of immune cells, which might be important pathophysiologic factors of diabetic nephropathy.

## Materials and Methods

### Data Acquisition

Single-cell RNA sequencing (ScRNA-seq) data of three early human diabetic kidney specimens and three controls were retrieved from the Gene Expression Omnibus (GEO) repository with accession number GSE131882 (https://www.ncbi.nlm.nih.gov/geo/query/acc.cgi?acc=GSE131882) (Wilson et al., 2019; Muto et al., 2021). This dataset was based on the GPL24676 Illumina NovaSeq 6000 platform. Two kidney transcriptomic profiling datasets (GSE111154 (Sircar et al., 2018) and GSE142025 (Fan et al., 2019)) were also acquired from the GEO repository. The GSE111154 dataset contained four normal kidneys and four early diabetic kidney samples on the platform of the GPL17586 Affymetrix Human Transcriptome Array 2.0 platform. Furthermore, the GSE142025 dataset included 28 diabetic kidney diseases and nine control samples on the basis of the GPL20301 Illumina HiSeq 4000 platform.

### Quality Control and Filtering of Cells

For droplet-based single-cell sequencing, usually, only data generated by droplets containing only one cell were retained. Unique molecular identifier (UMI) count matrix and the molecule information file (molecule_info.h5) generated by CellRanger were read by the DropletUtils package (version 1.14.2) (Lun et al., 2019), followed by the down-sampling of the UMI count matrix. The expression profile of each droplet and the expression profile of the surrounding solution were detected to distinguish between empty droplets that only had RNA in the solution and droplets containing cells. Then, the empty droplets were removed. Scater package (version 1.22.0) (McCarthy et al., 2017) was employed for further quality control. The total counts in the cell (library size), the number of genes containing counts in the cell, and the percentage of ribosome and mitochondrial genes were calculated by using the perCellQCMetrics function. The cells were filtered according to the percentage of mitochondrial genes ≤10%.

### Pre-Processing, Normalization and Dimensionality Reduction Analysis

NormalizeData function in Seurat package (version 4.1.0) (Satija et al., 2015) was utilized to standardize the expression matrix of each sample after filtering. By using the FindVariableFeatures function, the top 2,000 highly variable genes among cells were screened. ScaleData function was used for linearly scaling the expression data by converting the expression value of each gene so that the average expression value of each cell was 0 and the variance between cells was 1. Principal component analysis (PCA) was performed by the RunPCA function.

### Cell Clustering

FindNeighbors and FindClusters functions in Seurat package were utilized for cell clustering. The t-distributed stochastic neighbor embedding (t-SNE) and uniform manifold approximation and projection for dimension reduction (UMAP) were performed via RunTSNE and RunUMAP functions.

### Identification of Marker Genes and Cell Types

Using the FindMarkers function in the Seurat package (version 4.1.0), the differentially expressed genes were calculated in each cluster compared to all other clusters based on |log2fold-change| ≥0.1, the expression ratio of the cell population ≥0.25, and the *p*-value≤0.05, thereby obtaining marker genes. By comparing the marker genes with the existing marker genes, cell types were identified (Puthumana et al., 2021).

### Ligand-Receptor Network Analysis

Through the CellPhoneDB database (version 2.0) (Efremova et al., 2020), the cell–cell communication was inferred according to the expression values of the receptor ligands corresponding to diverse types of cells. Then, the ligand–receptor pairs between the cells were obtained. The ligand–receptor network was established by Cytoscape software (Shannon et al., 2003).

### Differential Expression Analyses and Protein-Protein Interaction (PPI)

The expression of marker genes that were obtained by the FindMarkers function was compared between diabetic kidney specimens and controls through the limma package (Ritchie et al., 2015). Differentially expressed marker genes with |log2fold-change|>1 and *p* < 0.05 were visualized into volcano plots and heat maps. The marker genes were imported into the STRING database (version 11.5) (von Mering et al., 2003). The PPI network was visualized via Cytoscape software (version 3.9.1).

### Functional Enrichment Analyses

Gene oncology (GO) enrichment analysis of marker genes was presented based on the gene oncology database (Ashburner et al., 2000), containing three categories: biological process, cellular component, and molecular function. Kyoto Encyclopedia of Genes and Genomes (KEGG) pathways enriched by marker genes were analyzed through the KEGG PATHWAY database (Kanehisa and Goto, 2000). *p*-value<0.05 was considered significant enrichment.

### Single-Cell Regulatory Network Inference and Clustering (SCENIC) Analysis

SCENIC algorithm was applied for reconstructing simultaneous gene regulatory networks and identifying cell state based on single-cell RNA-seq profiles (Aibar et al., 2017). According to the expression profiling of each cell, the area under the curve (AUC) value of each transcription factor corresponding to each cell was calculated to infer the gene set “activity” with the AUCell method. The AUC difference of each transcription factor between the diabetic nephropathy and control samples was compared with that of the limma package.

### Establishment of Diabetic Nephropathy Rat Model

Healthy Sprague–Dawley male rats (8–10 weeks old, weighing 200–250 g) were purchased from Beijing Huafukang Biotechnology Co., Ltd (China). All rats were reared in an environment of temperature (24 ± 0.5)°C, humidity (55 ± 5)%, and light/dark (12/12) h. After 1 week of adaptive feeding, they were randomly divided into a normal control group and a diabetic nephropathy model group. All rats had free access to water and food. The normal control group was given ordinary feed. The diabetic nephropathy model group was given high-fat and high-sugar feed. The feed formula was as follows: sucrose 20%, lard 10%, cholesterol 2.5%, sodium cholate 0.5%, and basic feed 67%. After 8 weeks of feeding, rats in the diabetic nephropathy model group were intraperitoneally injected with 30 mg/kg streptozotocin (Sigma, United States) solution dissolved in sodium citrate buffer, followed by continuing to feed on high-fat and high-sugar feed. After 7 days, blood was taken from the tail vein and the rat’s blood glucose was measured. Random blood glucose ≥16.7 mmol/L was the criterion for successful diabetes modeling. On this basis, the diabetic nephropathy model group was continuously given high-fat and high-sugar feed for 4 weeks and blood glucose was monitored. The 24-h urine albumin excretion rate was more than 20 mg and the urine glucose was positive, suggesting that the model of diabetic nephropathy was successful. All rats were anesthetized by an intraperitoneal injection of pentobarbital sodium (50 mg/kg). The kidneys of the rats were collected and washed with normal saline at 4°C. Finally, the kidney tissue was stored at -80 °C. This study gained the approval of the Ethics Committee of The First Affiliated Hospital of Harbin Medical University (2020-031).

### Hematoxylin-Eosin (H&E) Staining

The kidney tissue was fixed with 4% paraformaldehyde solution for 24 h. The tissue was dehydrated with gradient alcohol and xylene. Then, the tissue was embedded in wax. After drying, the tissue was cut into 4 μm thick sections. After patching, the dehydration step was reverse-performed as described previously. The section was stained by hematoxylin at room temperature for 5 min. Hydrochloric acid alcohol differentiation was presented. Afterwards, the section was stained by eosin for 30 s. Dehydrate transparent was carried out according to the previously mentioned dehydration step. Images were observed under a light microscope after mounting the slides.

### Real-Time Quantitative Polymerase-Chain Reaction (RT-qPCR)

Total RNA was extracted from 0.15 g kidney tissue through Trizol reagent (Solarbio, Beijing, China), followed by being reverse transcribed into cDNA. Using the SYBR green PCR kit (Toyobo, Japan) and the ABI Prism7300 fluorescence quantitative PCR instrument (ABI, United States), PCR amplification of the target genes was presented. The PCR amplification conditions were as follows: denaturation at 95 °C for 10 min, annealing at 60 °C for 1 min, and extension at 95°C for 15 s, 40 cycles. The primer sequences of target genes included: EIF4B, ATA​TCA​GTG​CAG​TGC​GTT​TA (F), and ATC​CCT​GTC​TTT​ATC​CTG​TG (R); RICTOR, GCATTTGGCAGTGGGTAT (F), and CCG​AGG​CTT​TAG​ATT​CAG​T (R); PRKCB, CCCAAAGACCCAAACTGA (F), and GCTGACGCTGCAACTTCT (R); GAPDH, GATGCTGGTGCTGAGTATGRCG (F), and GTGGTGCAGGATGCA TTGCTCTG (R). The 2^−ΔΔCt^ method was applied for semi-quantitatively analyzing the mRNA expression of the target genes.

### Western Blot

Kidney tissue was added to RIPA lysate (Thermo Fisher Scientific, United States) containing phosphatase inhibitor and protease, followed by being disrupted by an ultrasound. Then, the sample was centrifuged at 14,000 g at 4°C for 15 min, and the supernatant was collected. The BCA protein detection kit (Pierce, United States) was utilized for measuring the protein concentration. 20 μg protein was loaded. After 10% sodium dodecyl sulfate (SDS)-polyacrylamide gel electrophoresis, the product was transferred to a polyvinylidene fluoride (PVDF) membrane (Millipore, Germany). The membrane was blocked with 5% bovine serum albumin at 37°C for 2 h, and was incubated with primary antibodies overnight at 4°C. Antibodies included EIF4B (1/10000; ab134138; Abcam, United States), RICTOR (1/1000; ab219950; Abcam, United States), PRKCB (1/2000; ab195039; Abcam, United States), and GAPDH (1/10000; ab8245; Abcam, United States). After being washed 3 times with TBST, the membrane was incubated with HRP-labeled secondary antibody (1/2000; ab7097; Abcam, United States) for 2 h at 37°C. After being washed three times, the protein expression was detected by a gel quantitative analysis system following treatment with ECL (Solarbio, Beijing, China) with GAPDH as the reference control.

### Statistical Analyses

R language (version 3.5.2) and Graphpad Prism software (version 8.0.1) were employed for statistical analyses. Student’s t-test or one-way variance analyses were presented for comparisons between the groups. *p*-value<0.05 indicated statistical significance.

## Results

### Pre-Processing, Quality Control, and Normalization of scRNA-Seq Data of Diabetic Kidney Specimens and Controls

**FIGURE 1:**
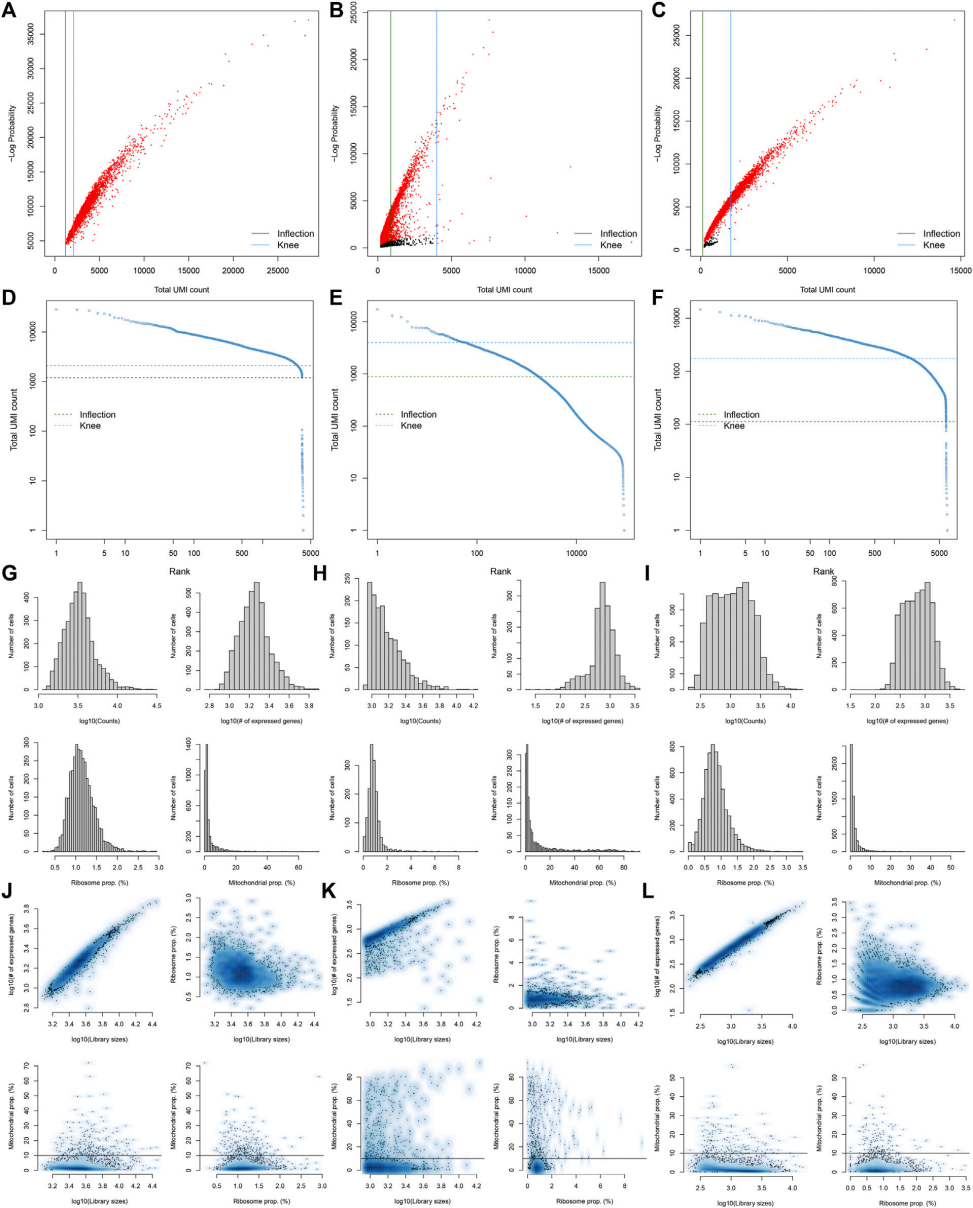
Pre-processing, quality control, and normalization of scRNA-seq data of the control kidney specimens. **(A–C)** The total count against the negative log-probability in three healthy kidney specimens. Red indicated cell droplet and black indicated empty droplet. **(D–F)** Barcode rank plots in three healthy kidney specimens. The inflection points of the knee indicate the transition of the total count distribution. **(G–L)** The ratios of mitochondrial and ribosomal genes expressed in each cell of the three healthy kidney specimens.

**FIGURE 2:**
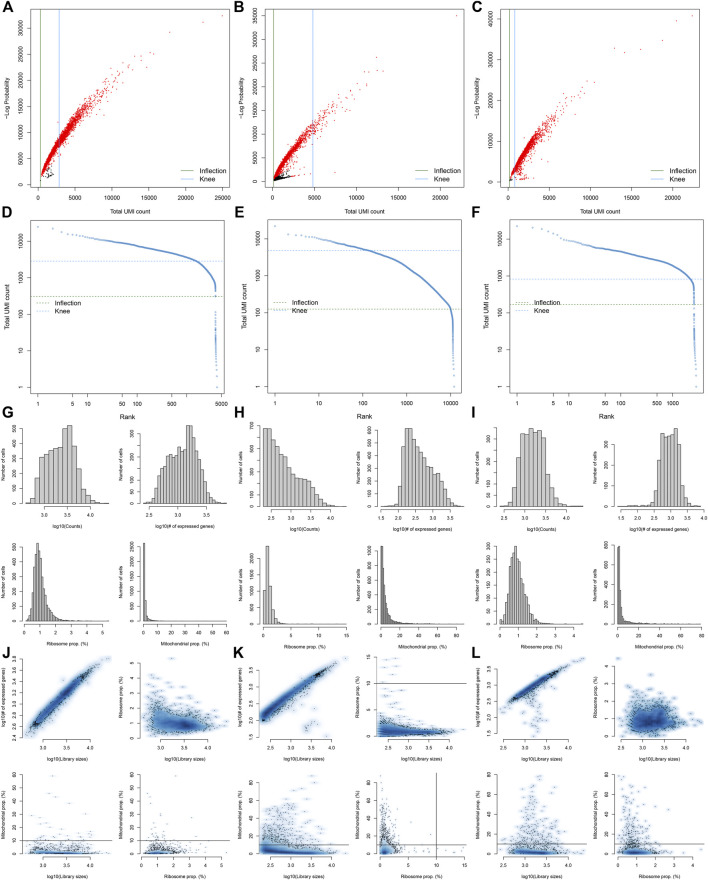
Pre-processing, quality control, and normalization of scRNA-seq data of the diabetic kidney specimens. **(A–C)** The total count against the negative log-probability in the three diabetic kidney specimens. Red indicated cell droplet and black indicated empty droplet. **(D–F)** Barcode rank plots in the three diabetic kidney specimens. The inflection points of the knee indicate the transition of the total count distribution. **(G–L)** The ratios of mitochondrial and ribosomal genes expressed in each cell of the three diabetic kidney specimens.

Here, we collected the scRNA-seq data of three early human diabetic kidney specimens and three controls from the GSE131882 dataset. Pre-processing, quality control, and normalization were firstly performed in each sample. The empty droplets may contain RNA from the surrounding solution, so counts were not zero. The emptyDrops function was employed for distinguishing empty droplets and cell droplets by examining the significant deviations between the expression profile of each barcode and the expression profile of the surrounding solution. As shown in Figures 1A–C, cell droplets had large negative log-probabilities and large total counts for three healthy kidney specimens. The empty droplet was then removed. The inflection points of the knee displayed the transition of the total count distribution in three healthy kidney specimens, reflecting the difference between empty droplets containing little RNA and cell droplets containing a large amount of RNA (Figures 1D–F). In each cell, the mitochondrial genome is only a small part compared to the nuclear genome. Therefore, the cells with a ratio of more than 10% of the mitochondrial genes were filtered. After filtering out cells with UMI <100, we calculated the proportion of mitochondrial and ribosomal genes expressed in each cell (Figures 1G–L). With the same methods, we performed pre-processing, quality control, and normalization of scRNA-seq data of the three diabetic kidney specimens (Figures 2A–L).

### Screening Highly Variable Genes and Cell Clustering

**FIGURE 3:**
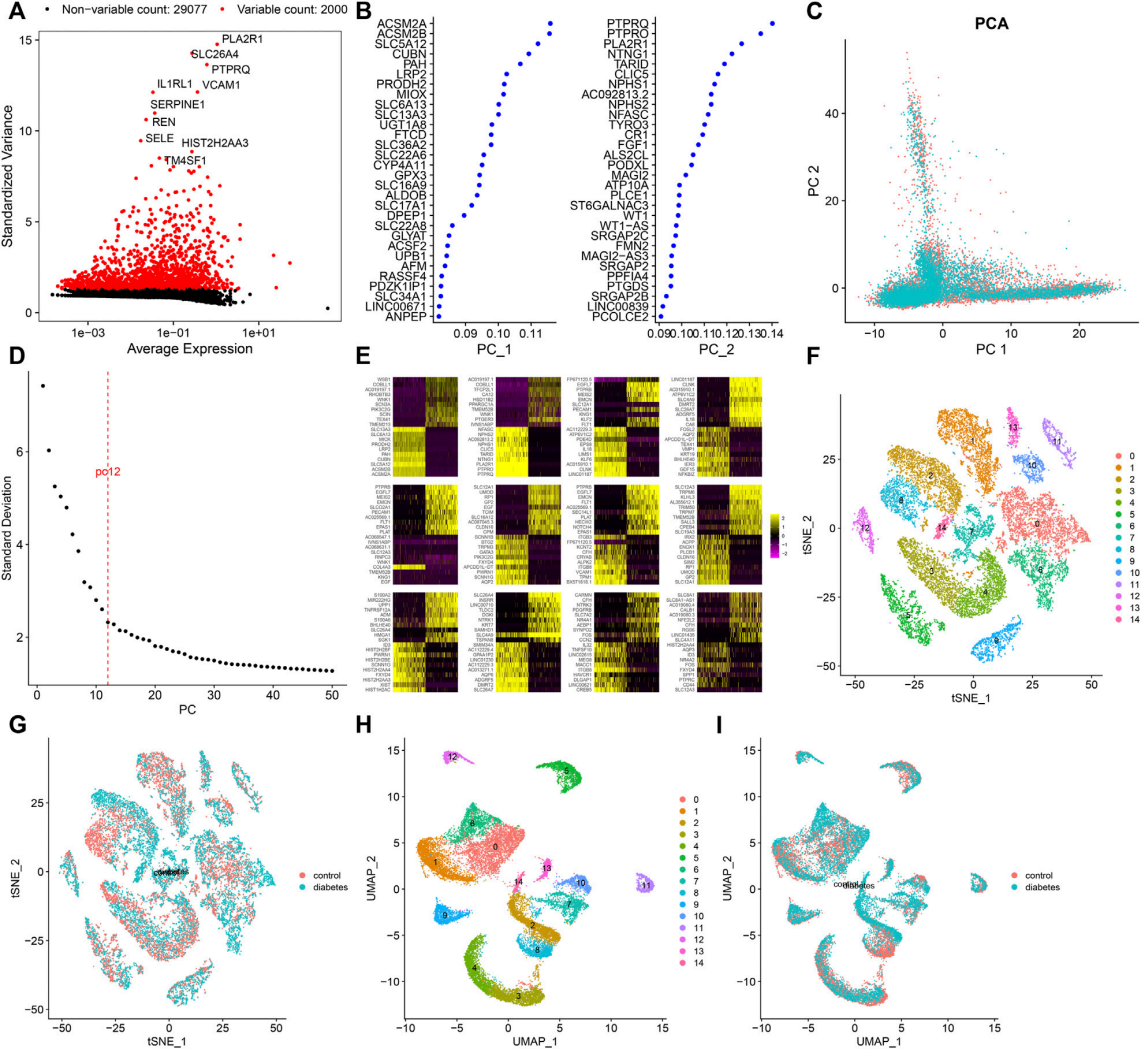
Screening highly variable genes and cell clustering. **(A)** Screening the top 2,000 highly variable genes across cells based on scRNA-seq data. Red dots indicate the highly variable genes and black dots indicate no significant genes. **(B)** Contribution of genes in the first two PCs. **(C)** PCA of filtered cells from the diabetic kidney specimens (green dots) and controls (red dots). **(D)** Elbow plot for identifying the optimal PCs. **(E)** Heat map visualizing the expression of marker genes in each PC. Cell clustering based on the **(F,G)** t-SNE and **(H,I)** UMAP methods.

**FIGURE 4:**
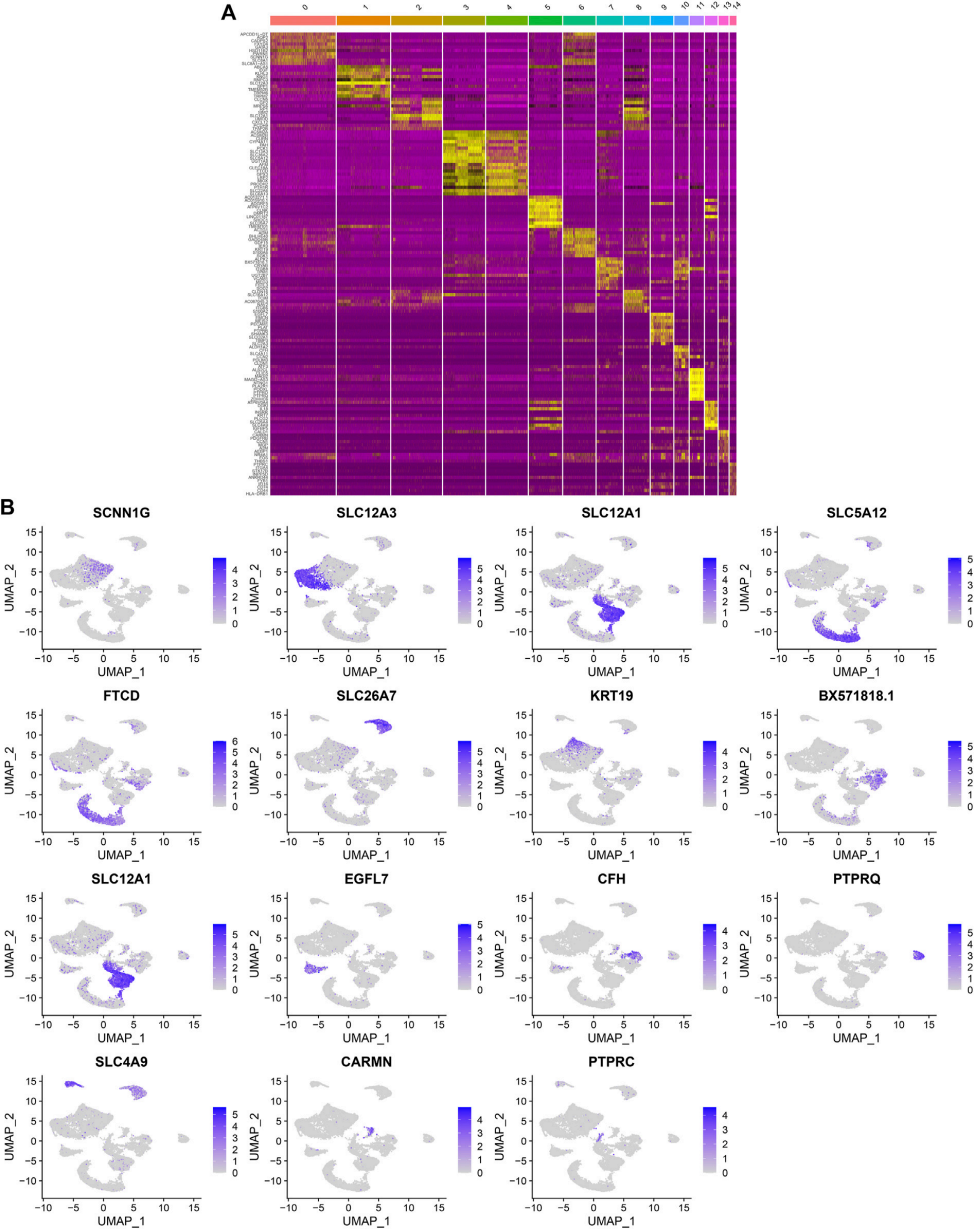
Identification of marker genes in each cell cluster. **(A)** Heat map showing the top ten marker genes in each cell cluster. Yellow represented high expression. **(B)** The expression distribution of the top one marker gene (SCNN1G, SLC12A3, SLC12A1, SLC5A12, FTCD, SLC26A7, KRT19, BX571818.1, EGFL7, CFH, PTPRQ, SLC4A9, CARMN, and PTPRC) in each cell cluster.

After standardizing the filtered scRNA-seq data, we selected the top 2,000 highly variable genes that showed large differences between cells, such as PLA2R1, SLC26A4, PTPRQ, IL1RL1, VCAM1, SERPINE1, REN, SELE, HIST2H2AA3, and TM4SF1 (Figure 3A). After scaling the data linearly with ScaleData function, we performed PCA on the scRNA-seq data for the dimensionality reduction analysis using the RunPCA function (Figures 3B,C). The Elbow plot ranked the main components based on the percentage of variance revealed by each component. We found that the elbow appeared when PC = 12, indicating that most of the real signals were captured in the first 12 PCs (Figure 3D). Heat maps visualized the marker genes in the first 12 PCs (Figure 3E). By applying two methods, t-SNE and UMAP, we presented the cell clustering analysis. As a result, 12 cell clusters were identified across diabetic kidney specimens and controls (Figures 3F–I). Using the FindMarkers function, we found the differentially expressed genes in each cell type compared to other types, as the marker genes of the cell type. As shown in Figure 4A, the top ten marker genes were identified in each cell cluster. Moreover, we showed the top one marker genes in each cell cluster, including SCNN1G, SLC12A3, SLC12A1, SLC5A12, FTCD, SLC26A7, KRT19, BX571818.1, EGFL7, CFH, PTPRQ, SLC4A9, CARMN, and PTPRC (Figure 4B).

### Identification of Cell Types and Their Marker Genes

**FIGURE 5:**
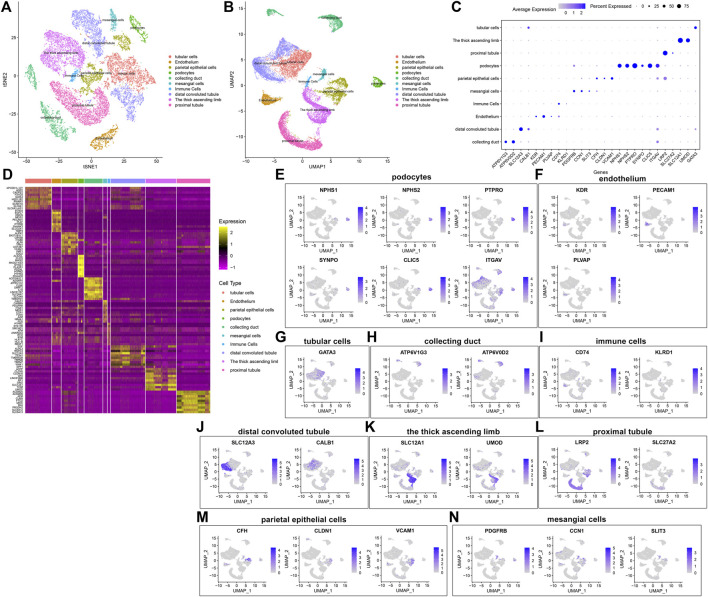
Identification of cell types and their marker genes. **(A,B)** Identification of cell types in the diabetic kidney specimens and controls. Each cell type was marked by a unique color. **(C)** The expression patterns of marker genes among cell types **(D)** Heat map for the top ten marker genes in each cell type. The expression distribution of marker genes in **(E)** podocytes, **(F)** endothelium, **(G)** tubular cells; **(H)** collecting duct; **(I)** immune cells; **(J)** distal convoluted tubule; **(K)** the thick ascending limb; **(L)** proximal tubule; **(M)** parietal epithelial cells; and **(N)** mesangial cells.

By comparing the marker genes that we identified with the published cell-type-specific markers, we defined the cell groups that we clustered, including tubular cells, endothelium, parietal epithelial cells, podocytes, collecting duct, mesangial cells, immune cells, distal convoluted tubule, the thick ascending limb, and proximal tubule (Figures 5A,B). Marker genes were identified in each cell type by comparing with other cell types. Figure 5C depicts the expression patterns of marker genes among the cell types. A heat map showed the top ten marker genes in each cell type (Figure 5D). We further visualized the expression distribution of marker genes in each cell type as follows: NPHS1, NPHS2, PTPRO, SYNPO, CLIC5, and ITGAV in podocytes (Figure 5E); KDR, PECAM1, and PLVAP in endothelium (Figure 5F); GATA3 in tubular cells (Figure 5G); ATP6V1G3 and ATP6V0D2 in collecting duct (Figure 5H); CD74 and KLRD1 in immune cells (Figure 5I); SLC12A3 and CALB1 in distal convoluted tubule (Figure 5J); SLC12A1 and UMOD in the thick ascending limb (Figure 5K); LRP2 and SLC27A2 in proximal tubule (Figure 5L); CFH, CLDN1, and VCAM1 in parietal epithelial cells (Figure 5M); and PDGFRB, CCN1, and SLIT3 in mesangial cells (Figure 5N).

### Establishment of a Ligand-Receptor Network

**FIGURE 6:**
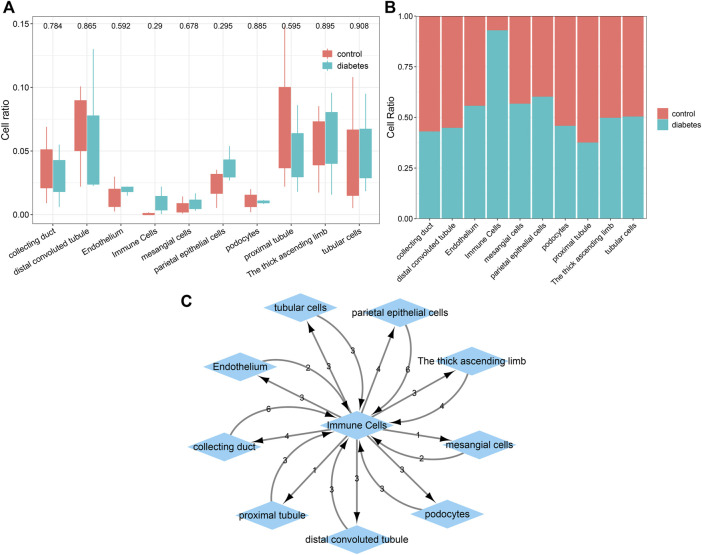
Comparisons of the difference in various cell types between diabetic kidney specimens and controls and the establishment of a ligand–receptor network. **(A)** Box plots showing the differences in the cell ratios of collecting duct, distal convoluted tubule, endothelium, immune cells, mesangial cells, parietal epithelial cells, podocytes, proximal tubule, the thick ascending limb, and tubular cells between the diabetic kidney specimens and controls. **(B)** Stacked graph for the cell ratios among the aforementioned cell types both in diabetic kidney specimens and controls. **(C)** The ligand–receptor network among the aforementioned cell types. The number represents the number of relationship pairs.

The percentages of cells were compared between the diabetic kidney specimens and controls. We found that the collecting duct, distal convoluted tubule, podocytes, and proximal tubule exhibited decreased percentages in the diabetic kidney specimens compared to those in the controls (Figure 6A). Meanwhile, endothelium, immune cells, mesangial cells, parietal epithelial cells, the thick ascending limb, and tubular cells had increased percentages in the diabetic kidney specimens than in the controls. As shown in Figure 6B, immune cells had the highest cell ratio among all cell types in the diabetic kidney specimens, and the opposite results were found in control specimens, indicating that immune cells could play a prominent role in diabetic nephropathy progression. Cell–cell communication mediated by ligand–receptor complexes plays key roles in coordinating diverse biological processes. By CellPhoneDB v2.0, we identified receptor–ligand pairs between cells. Figure 6C shows that immune cells had close interactions with other cells by receptor–ligand interactions. Supplementary Table S1 listed the receptor–ligand pairs of differentially expressed marker genes of immune cells.

### Identification of Differentially Expressed Marker Genes of Immune Cells in Diabetic Nephropathy

**FIGURE 7:**
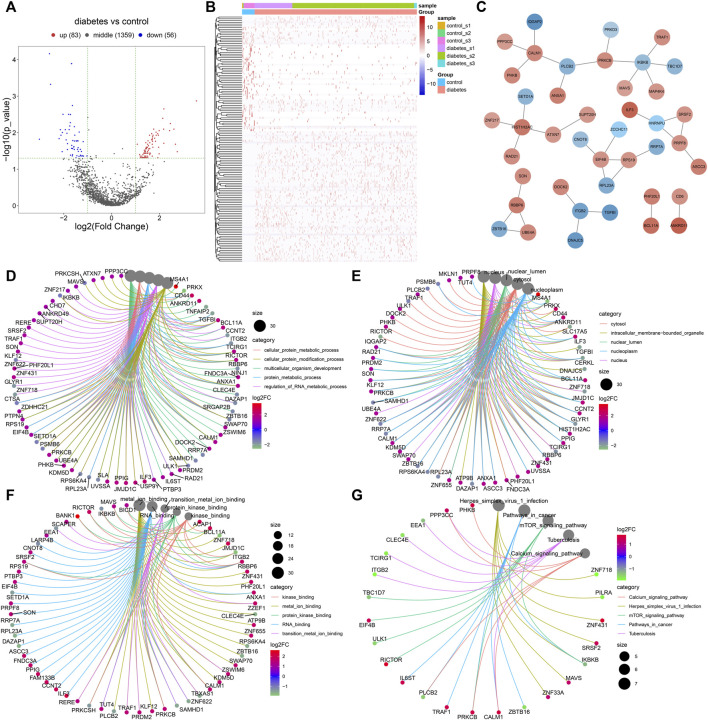
Identification of differentially expressed marker genes of immune cells in diabetic nephropathy and analysis of their biological implications. **(A)** Volcano diagram showing the expression differences in marker genes of immune cells between the diabetic kidney specimens and controls. Blue: down-regulation; red: up-regulation and grey: no significance. **(B)** Hierarchical clustering analysis visualizing the expression patterns of differentially expressed marker genes of immune cells between diabetic kidney specimens and controls. Blue indicates down-regulation and red indicates up-regulation. **(C)** The PPI network based on the differentially expressed marker genes of immune cells. The depth of the color of bubble was proportional to |log2fold-change|. Blue indicated down-regulation and red indicated up-regulation. **(D)** Biological processes, **(E)** cellular components, **(F)** molecular functions, and **(G)** KEGG pathways enriched by the differentially expressed marker genes of immune cells. The size of the bubble was proportional to the number of enriched genes.

With |log2fold-change|>1 and *p* < 0.05, we identified 83 up-regulated and 56 down-regulated marker genes of immune cells in the diabetic kidney specimens compared to the controls (Figure 7A,B; Supplementary Table S2). The PPI network revealed the tight interactions between these differentially expressed marker genes of immune cells (Figure 7C). IKBKB (degree = 5), HIST1H2AC (degree = 4), EIF4B (degree = 4), and CALM1 (degree = 4) had the highest degree, which were considered as hub genes. The biological implications of the differentially expressed marker genes of immune cells were further analyzed. In Figure 7D, these marker genes were markedly involved in cellular protein metabolic process, cellular protein modification process, multi-cellular organism development, protein metabolic process, and regulation of RNA metabolic process. They significantly participated in regulating cellular components of cytosol, intracellular membrane-bounded organelle, nuclear lumen, nucleoplasm, and nucleus (Figure 7E). As shown in Figure 7F, the marker genes had the molecular functions of kinase binding, metal ion binding, protein kinase binding, RNA binding, and transition metal ion binding. Moreover, calcium signaling pathway, herpes simplex virus one infection, mTOR signaling pathway, pathway in cancer, and *tuberculosis* were significantly enriched by the marker genes (Figure 7G). The aforementioned findings indicated the important biological functions of the differentially expressed marker genes of immune cells.

### Single-Cell Regulatory Network Analysis

**FIGURE 8:**
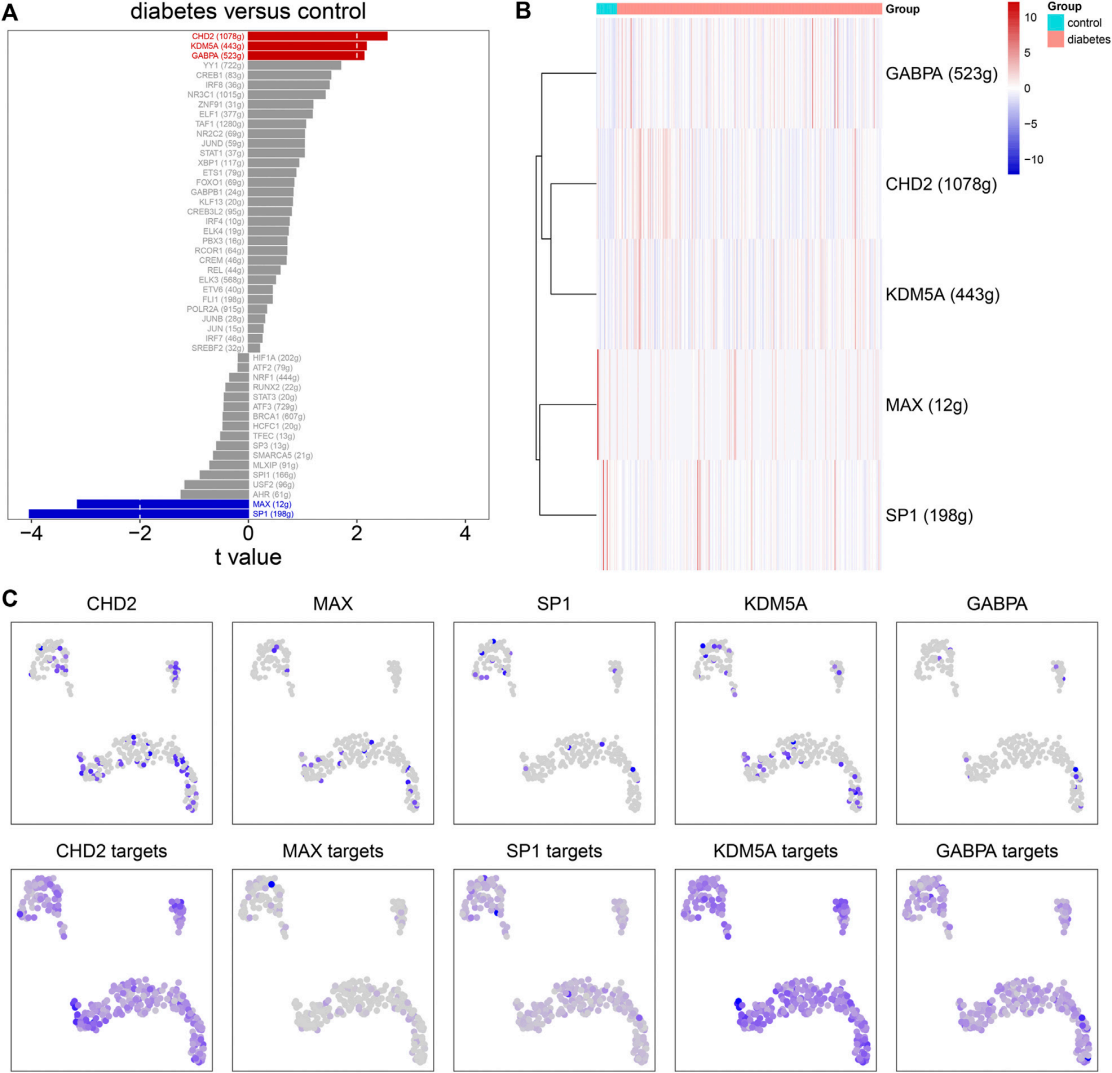
Single-cell regulatory network analysis. **(A)** The top 50 transcription factors according to the t values by comparing AUC values between diabetic nephropathy and controls. **(B)** Heat map for the AUC values of the main transcription factors in diabetic kidney specimens compared to controls. **(C)** The expression levels (upper) and AUC values (bottom) of the main transcription factors in immune cells.

SCENIC was applied to infer the AUC values of each transcription factor corresponding to each cell on the basis of the expression profiles of each cell. The AUC was used to reveal whether a key subset of the input gene set was enriched in the expressed genes of each cell. By clustering cells by this regulator activity, we can find whether there were cell populations that tended to have the same regulator activity, and reveal the network state that occurred repeatedly in multiple cells. By using the limma package, we calculated the AUC difference of each transcription factor between diabetic nephropathy and controls. Figure 8A lists the top 50 transcription factors according to the t values. Among them, GABPA, CHD2, KDM5A, MAX, and SP1 were considered as the main transcription factors. Compared to controls, GABPA, CHD2, and KDM5A had higher AUC values.MAX and SP1 had lowered AUC values in diabetic nephropathy (Figure 8B). Immune cells had the high regulation activity of CHD2, MAX, SP1, KDM5A, and GABPA based on scRNA-seq data (Figure 8C).

### Validation of the Expression of mTOR Pathway Markers in External Datasets

**FIGURE 9:**
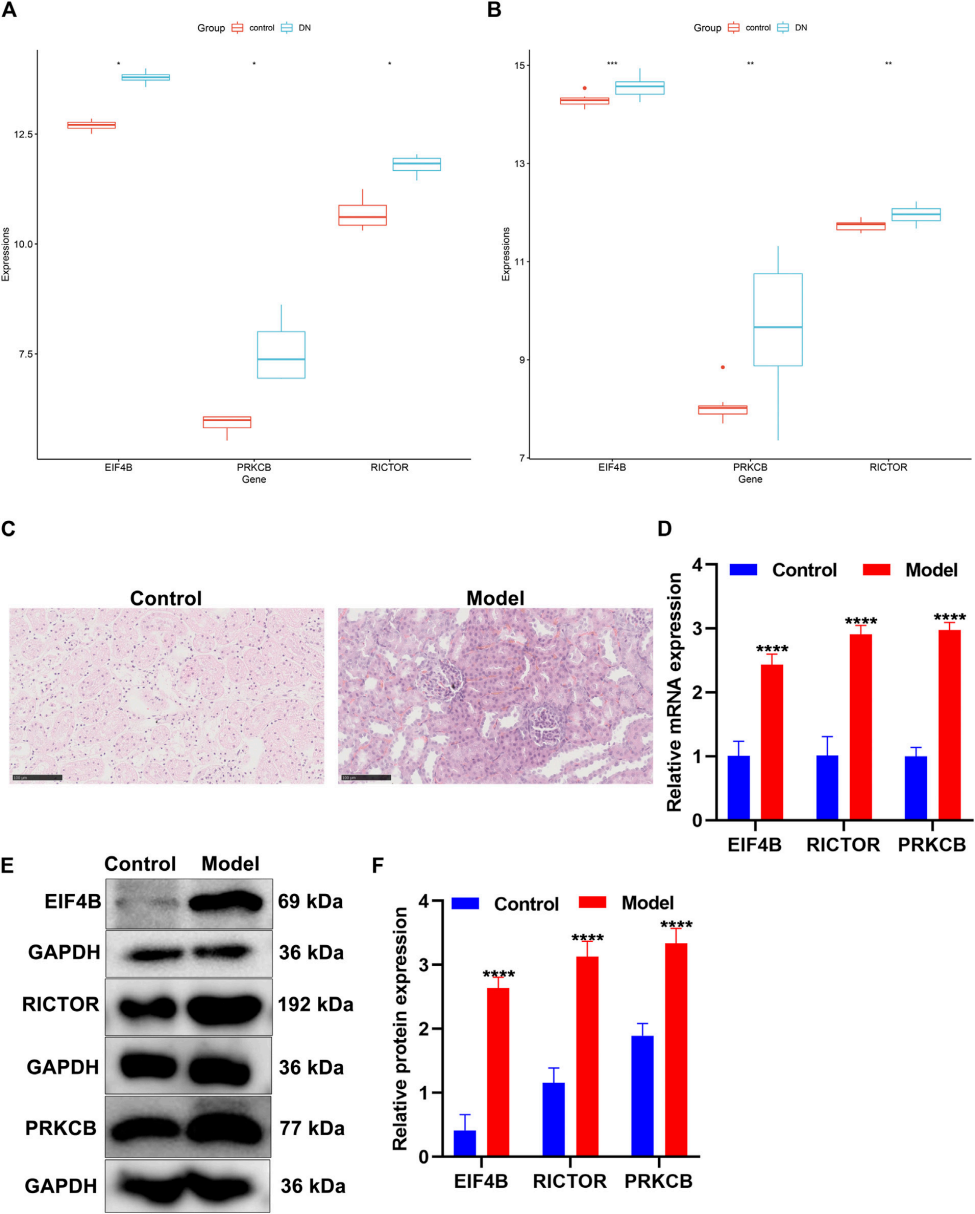
Validation of the expression of the mTOR pathway markers in diabetic nephropathy. **(A,B)** Box plots of the expression of mTOR pathway markers EIF4B, RICTOR, and PRKCB in control and diabetic nephropathy samples in the **(A)** GSE111154 and **(B)** GSE142025 datasets. DN: diabetic nephropathy. **(C)** H&E staining of the morphology of kidney tissues in the control group and the diabetic nephropathy rat model group. Scale bar, 100 μm; magnification, ×400. **(D)** The mRNA expression of EIF4B, RICTOR, and PRKCB was determined in kidney tissues from the control and diabetic nephropathy groups through RT-qPCR. **(E,F)** The expression of EIF4B, RICTOR, and PRKCB proteins was determined in kidney tissues from the control and diabetic nephropathy groups through Western blot. *p < 0.05; **p < 0.01; ***p < 0.001; ****p < 0.0001.

Accumulated evidence has confirmed the crucial role of the mTOR pathway in diabetic nephropathy (Qiao et al., 2019). We found that differentially expressed marker genes (EIF4B, RICTOR, and PRKCB) of immune cells were enriched in the mTOR pathway, indicating that these marker genes could participate in mediating the pathway. The GSE111154 and GSE142025 datasets were utilized for validating the expression of mTOR pathway markers EIF4B, RICTOR, and PRKCB in the kidneys from controls and diabetic nephropathy patients. As expected, their expression was significantly up-regulated in diabetic nephropathy compared with controls both in the GSE111154 (Figure 9A) and GSE142025 (Figure 9B) datasets.

### Construction of Diabetic Nephropathy Rat Models

After streptozotocin-induced diabetic rats were fed with a high-fat and high-sugar diet for 4 weeks after being modeled, the rats in the model group showed significant polydipsia, polyphagia, polyuria, weight loss, listlessness, and hair exhaustion, indicating that the rats in the model group had already developed obvious symptoms of diabetic nephropathy. H&E staining showed that the structure of the rat kidney in the control group was neat and clear, and the glomerular morphology, the renal tubules, and the cytoplasmic staining were normal (Figure 9C). Compared with the control group, the renal tubule lumen of the diabetic nephropathy model group became larger, with hyaline casts visible in the lumen with obvious vacuolization, lighter cytoplasmic staining, and pyknosis of renal tubular epithelial cells (Figure 9C). The glomerulus became smaller, the mesangium was enlarged, the basement membrane was thickened, and the nucleus pyknosis was obvious.

### Verification of the Expression of mTOR Pathway Markers in Diabetic Nephropathy Rat Models

The expression of mTOR pathway markers EIF4B, RICTOR, and PRKCB was verified in kidney tissues from the control group and the diabetic nephropathy rat model group. Our data showed that in comparison to the control group, EIF4B, RICTOR, and PRKCB displayed markedly higher mRNA expression in diabetic nephropathy rats (Figure 9D). Meanwhile, Western blot results confirmed the significant up-regulation of EIF4B, RICTOR, and PRKCB proteins in kidney tissues of diabetic nephropathy rats than that of control rats (Figures 9E,F). This confirmed the activation of the mTOR pathway in diabetic nephropathy.

## Discussion

ScRNA-seq allows observing gene expression changes on the cell-by-cell basis. Through scRNA-seq, we identified highly variable genes that strongly cause variations between cells across homogeneous cell populations (Cao et al., 2020). Identification of cell types is a common method to interpret scRNA-seq data and resolve cellular heterogeneity based on the interactions between the transcriptomes and phenotypes (Wang et al., 2020). By scRNA-seq profiles, we identified specific cell types and their marker genes in diabetic nephropathy, which might assist in finding new treatments for diabetic nephropathy.

Herein, this study identified 10 cell types in diabetic nephropathy tissues and controls, including tubular cells, endothelium, parietal epithelial cells, podocytes, collecting duct, mesangial cells, immune cells, distal convoluted tubule, the thick ascending limb, and proximal tubule in diabetic kidney specimens and controls. Among them, immune cells had the highest proportion in diabetic nephropathy, indicating that immune cells played a prominent role in the progression of diabetic nephropathy. Epidemiological and preclinical evidence suggests the relationships between inflammation and diabetic nephropathy pathogenesis. Targeting inflammation such as anti-inflammatory mediators and anti-intracellular pathways has showed favorable therapeutic effects in experimental diabetic nephropathy models, such as reducing proteinuria and renal lesions (Rayego-Mateos et al., 2020).

The mTOR pathway participates in the pathogenesis of diabetic nephropathy (Ma et al., 2018). Inhibiting the mTOR pathway can prevent the early structural alterations in the renal tissues developing towards diabetic nephropathy (Ma et al., 2018). Here, our study found that marker genes of immune cells EIF4B, RICTOR, and PRKCB were significantly enriched in the mTOR pathway, which were significantly up-regulated in the diabetic kidney specimens than in the controls. To verify the expression of EIF4B, RICTOR, and PRKCB proteins, we established a diabetic nephropathy rat model. Our RT-qPCR and Western blot results confirmed the significant up-regulation of EIF4B, RICTOR, and PRKCB in diabetic kidney specimens. Consistently, a previous study has detected the significant overexpression of RICTOR in renal tissues of diabetic nephropathy rat models (Wang et al., 2019). MiRNA-424 facilitates diabetic nephropathy progression through targeting RICTOR both in diabetic nephropathy rat models and high-glucose–induced glomerular mesangial cell models (Wang et al., 2019). Inhibiting RICTOR could alleviate high-glucose–induced podocyte apoptosis (Song et al., 2020). PRKCB is markedly up-regulated in kidney biopsies of diabetic patients (Langham et al., 2008). Polymorphisms of the PRKCB1 protein may facilitate kidney diseases in type 2 diabetes (Araki et al., 2006). Collectively, marker genes of immune cells EIF4B, RICTOR, and PRKCB in the mTOR pathway might participate in diabetic nephropathy progression.

Several limitations of our study should be acknowledged. Although the expression of EIF4B, RICTOR, and PRKCB was verified in a diabetic nephropathy rat model, a deeper analysis should be conducted for confirming their functions in diabetic nephropathy progression.

## Conclusion

Collectively, our study identified 10 specific cell types and their marker genes in diabetic nephropathy. Among all cell types, immune cells had the highest ratio in diabetic nephropathy tissues, which were considered as a determinant during diabetic nephropathy progression. Also, we established diabetic nephropathy rat models; marker genes (EIF4B, RICTOR, and PRKCB) of the immune cells were confirmed to be up-regulated in diabetic nephropathy. Thus, immune cells and their marker genes might be important pathophysiologic factors of diabetic nephropathy.

## Abbreviations

AUC, area under the curve; GO, Gene oncology; H&E, Hematoxylin-eosin; KEGG, Kyoto Encyclopedia of Genes and Genomes; PPI, protein-protein interaction; RT-qPCR, Real-time quantitative polymerase-chain reaction; SCENIC, single-cell regulatory network inference and clustering; RNA-seq, RNA sequencing; scRNA-seq, single-cell RNA sequencing; GEO, Gene Expression Omnibus; UMI, unique molecular identifier; PCA, principal component analysis; t-SNE, t-distributed stochastic neighbor embedding; UMAP, uniform manifold approximation and projection.

## Data Availability

The original contributions presented in the study are included in the article/Supplementary Material, further inquiries can be directed to the corresponding author.

## Abbreviations

AUC, area under the curve; GO, Gene oncology; H&E, Hematoxylin‐eosin; KEGG, Kyoto Encyclopedia of Genes and Genomes; PPI, protein‐protein interaction; RT‐qPCR, Real‐time quantitative polymerase‐chain reaction; SCENIC, single‐cell regulatory network inference and clustering; RNA‐seq, RNA sequencing; scRNA‐seq, single‐cell RNA sequencing; GEO, Gene Expression Omnibus; UMI, unique molecular identifier; PCA, principal component analysis; t‐SNE, t‐distributed stochastic neighbor embedding; UMAP, uniform manifold approximation and projection.

## Supplementary Material

Supplementary Table S1 Receptor-ligand pairs of differentially expressed marker genes of immune cells.

Supplementary Table S2 Differentially expressed marker genes of immune cells in diabetic kidney specimens compared to controls.
